# Early removal of etonogestrel subcutaneous contraceptive implant at a community health centre in Pretoria

**DOI:** 10.4102/safp.v64i1.5407

**Published:** 2022-07-07

**Authors:** Dikonketjo M.P. Moeti, Indiran Govender, Tombo Bongongo

**Affiliations:** 1 Department of Family Medicine and Primary Health Care, Faculty of Health Sciences, Sefako Makgatho Health Sciences University, Pretoria, South Africa

**Keywords:** early removal, etonogestrel, subcutaneous contraceptive, implant, Pretoria, community health centre, weight gain, vaginal bleeding

## Abstract

**Background:**

The etonogestrel subcutaneous contraceptive implant offers efficacy for three years, but some women remove it earlier than prescribed. This study discusses factors associated with the early removal of these implants at a Pretoria community health centre between 01 January 2020 to 30 June 2020.

**Methods:**

A cross-sectional study using a piloted and researcher assistant-administered questionnaire.

**Results:**

Of the 124 participants who removed their etonogestrel subcutaneous contraceptive implant earlier than prescribed, most were single, unemployed, in the age group 30–39 years, Christian, with secondary level education and with parity one or more. Etonogestrel subcutaneous contraceptive implant pre-insertion counselling was given to all participants, most of whom had not previously used contraceptives. Those participants with previous contraceptive use had used injectables. Long-term contraception was the main reason for getting the etonogestrel subcutaneous contraceptive implant. Most participants did not attend post-insertion counselling. Heavy bleeding was the most common side effect and reason for early removal. Fifty-one participants kept the etonogestrel subcutaneous contraceptive implant in for a longer period of 12–23 months. From participants’ responses, it seems that Etonogestrel implants may be offered from as early as 15–20 years of age.

**Conclusion:**

Women having etonogestrel subcutaneous contraceptive implants removed early at a Pretoria community health centre tended to be young, single, unemployed, Christian, with a secondary level education and with parity one or more. All participants attended the etonogestrel subcutaneous contraceptive implant pre-insertion counselling services but not the post-counselling services. Heavy bleeding was the main reason for the early removal of the etonogestrel subcutaneous contraceptive implant.

## Introduction

Contraception is defined as any method, device (chemical, mechanical or surgical) or sexual practice used to prevent pregnancy.^1^ There are different types of contraceptive measures; this study focused on the etonogestrel subcutaneous contraceptive implant. This is a single rod-shaped subcutaneous contraceptive implant containing 68 mg etonogestrel, with effectiveness of up to three years. It is placed in the inner groove of the upper arm, about 8 cm – 10 cm above the medial epicondyle of the humerus.^2^ It affects contraception by inhibiting ovulation, as well as by thickening the cervical mucus. It is a convenient, cost-effective and efficient contraceptive choice, requiring about three visits to the healthcare provider in three years. The first visit is for insertion of the contraceptive subdermally by a trained healthcare professional, the second is for a 3-month check-up post-insertion, and the third visit at the end of the three years would be for removal of the implant.^2^

The American College of Obstetricians and Gynaecologists (ACOG) recommends that long-acting reversible contraceptives (LARCs) should be used as the first line of contraception and inserted on the same day that the patient presents to the healthcare facility.^3^ This includes immediately after an abortion or giving birth.^3^

Teenage pregnancy leading to poor health outcomes for both teenage mothers and their offspring remains a global public health concern.^4^ The ACOG addressed this by recommending etonogestrel subcutaneous contraceptive implant placement for teenagers and young adult females.^5^ The etonogestrel subcutaneous contraceptive implant is an effective form of contraception, specifically for the noncompliant adolescent who is sexually active with multiple partners, as it does not rely on user adherence for effectiveness.^6^ Young women, first-time contraceptive users, women who have just given birth (immediately postpartum) and women who have just had abortions should be targeted and prioritised for use of LARCs.^7^

Research has established that American and Asian women prefer long-term methods of contraception, such as the etonogestrel subcutaneous contraceptive implant and the intrauterine contraceptive device (IUCD).^8^ In South Africa, the use of modern contraceptives is still low, especially amongst disadvantaged young women living in rural areas. The most common contraceptive method used in the country is the injectable, followed by the oral pill.^9,10^

A study conducted in KwaZulu-Natal (KZN) in South Africa which aimed at describing the reasons for requesting removal of the etonogestrel subcutaneous contraceptive implant found about 24.2% of participants requested its removal before three years were up. In 2018, in East London, South Africa, it was found that of 188 women who participated in a study, the majority (67.3%) had removed the implant in the first year of use, whilst 94.4% had removed it after the second year. The average duration of use was 11 months.^11^

Most women (82.8%) who discontinued the etonogestrel subcutaneous contraceptive implant early cited side effects as the reason.^10^ Prolonged, heavy or irregular menstrual bleeding was the most common side effect mentioned.^10,11^ In the KZN study, side effects such as heavy menstrual bleeding, severe headache and painful arm were cited by 71.3% of respondents as causes for early removal of the etonogestrel subcutaneous contraceptive implant.^11^ Other reasons for discontinuation that were mentioned included dissatisfaction with the positioning of the implant (3.2%), a desire to fall pregnant (4.3%) and the use of chronic medication.^11^ Research has shown that the etonogestrel subcutaneous contraceptive implant interacts with efavirenz, rifampicin and anti-epileptics, reducing their effectiveness. Because of this, 12.8% of early implant discontinuation is in women recently diagnosed with human immunodeficiency virus (HIV) and who are on antiretrovirals (ARVs).^10,12,13^

A large number of women in South Africa who are using the etonogestrel subcutaneous contraceptive implant do not know their HIV status and are not aware of issues such as the etonogestrel subcutaneous contraceptive implant’s interaction with chronic medications such as ARVs.^10^ Efavirenz is known to lower the efficacy of the subcutaneous contraceptive implant by lowering serum levels of etonogestrel.^13^ However, the implant still appears to be the most effective contraceptive in women taking ARVs.^13^

A study carried out in Botswana in 2020 found that the implant remained a highly effective contraception option for women living with HIV who use a regimen that contains dolutegravir.^14^ High levels of satisfaction regarding the use of the implant were found amongst most of the women, who used it for its full duration, and most chose to have it reinserted when the prescribed term of use of three years was over. This showed satisfaction with and acceptability of this method amongst these women.^12^

In family planning counselling, contraceptive side effects and the choice of contraceptive method are often not well addressed.^15^ Studies have shown that LARCs such as IUCDs and implants are given priority by women when they are well informed regarding these contraceptive methods and if they are provided free of charge.^16^ In 2016, only about 4% of the sexually active female population in South Africa was using the implant.^17^ It was discovered that most of the women using the implant had heard about it from acquaintances by word of mouth and had gone to the clinic themselves to request it.^10^ Only about 7% of South African women using the implant heard about it from publicity material, at schools or clinics or on the internet, television, or social media, whilst about 30% had heard about the implant from a healthcare provider.^10^

Society and the community play a major role in influencing women’s perception of contraception, and hence there is a need to address any misperceptions about the implant and to promote accurate information about it in the community.^18^ The utilisation of step-by-step diagrams and flow charts in consultation rooms that constantly remind one of the correct processes to follow was suggested to improve the quality and efficacy of the preinsertion counselling service.^10^

In Ethiopia, a positive association has been shown between follow-up appointments after implant insertion and decreased implant discontinuation rates.^7,19,20^ It was found that 65% of women who had follow-up appointments after insertion were less likely to discontinue the implant. It is suggested that counselling and continued support, including treatment, from healthcare professionals during the follow-up appointments regarding side effects such as menstrual abnormalities contribute to reduced discontinuation rates.^20^

The World Health Organization (WHO) has declared that routine follow-up of women who have had an implant inserted is not needed.^21^ This is in contrast to the advice from Merck, which recommended to review women three months after insertion of implant as a check-up, as discomforts such as bleeding abnormalities occur more often, as highlighted in the literature, and can be the main reason for early discontinuation of the implant.^9^

Despite these recommendations, the WHO and the South African Department of Health state that patients should come for follow-up at their own discretion.^21,22^ The 3-month follow-up recommendation by Merck is currently not part of policy in South Africa.^2,22^

Although the implant seems to be presented as an effective contraceptive^2^ and prioritised by women because of its efficacy,^18^ quite a number of women attending a Pretoria community health centre (CHC) removed it earlier than prescribed (or before 2.5 years). This behaviour motivated the researcher to carry out this study, which focuses on factors associated with the early removal of etonogestrel subcutaneous contraceptive implant at a CHC in the Pretoria.

## Methods

### Study design

This was a quantitative descriptive study with a cross-sectional design. A piloted and researcher assistant-administered questionnaire was used amongst women with a history of early removal of the etonogestrel subcutaneous contraceptive implant, seen at Soshanguve 3 CHC, Pretoria.

### Data collection

The researcher trained a retired nurse who used to work in the family planning unit at Soshanguve 3 CHC. She was able to express herself fluently in both English and Setswana, the two most widely spoken languages in the study area. She was tasked with explaining the aim and objectives of the study to participants of child-bearing age who could consent for themselves and requested early implant removal. The questionnaire, written in English and in Setswana, was administered by the research assistant in the preferred language of the participant. Signed written consent was a requirement before enrolling a participant. Each completed questionnaire was marked with a number (1, 2, 3, etc.) and the same number was written on the first page of the file of each participant in order to avoid recruiting the same participant more than once.

### Study setting

The study was conducted in a CHC (Soshanguve 3 CHC) located in a township (semi-rural zone) named Soshanguve that is about 30 km north of Pretoria, Gauteng, South Africa.

### Study population and sampling

From the implant removal register of Soshanguve 3 CHC, an average of five women per week remove the implant. In the 6-month period (from 01 January 2020 to 30 June 2020) which was allocated for data collection, roughly 120 women were expected to remove their implants. Convenience sampling applied, all women who requested early implant removal were approached to take part in the study, and those who consented to do so were recruited for the sample. At the end of data collection, there was oversampling, and the total number of women included was 124 (*n* = 124).

### Data analysis

Raw data were captured on an Excel spreadsheet and then imported into Statistical Analysis System (SAS) version 9.4, where statistical analyses were performed. Associations of variables were assessed using Fisher’s exact test, with a *p*-value of less than or equal to 0.01 denoting significance. The results are presented in tables in the form of frequencies and percentages.

### Ethical considerations

Ethical clearance to conduct this study was obtained from the Sefako Makgatho University Research Ethics Committee (number: SMUREC/M/224/2019: PG). Research clearance was obtained from the Tshwane Research Committee of the Gauteng Department of Health (number: GP_201910_032), and permission was provided by the district authority (National Human Resource Development [NHRD] reference number: GP_201910-022). Confidentiality was maintained throughout the study process. Signed written consent was a requirement before enrolling a participant. Participants were told that they could withdraw from the study at any time during the study process if they were uncomfortable, and such withdrawal would not affect their health care. However, no participants withdrew from the study.

## Results

The majority of participants were from the age group 18–29 years, had a secondary level of education, were single, unemployed and had children, as presented in Table 1.

**TABLE 1:** Sociodemographic characteristics of the sample.

Characteristic	Frequency (*n*)	Percentage
**Age (years)**		
18–29	100	80.65
30–39	19	15.32
40–49	5	4.03
**Religion**		
Christian	117	94.35
Non-Christian	7	5.65
**Education levels**		
Primary	7	5.65
Secondary	78	62.90
Tertiary	39	31.45
**Marital status**		
Single	100	80.65
Married	24	19.35
**Employment**		
Yes	47	37.90
No	77	62.10
**Have children**		
Yes	81	65.32
No	43	34.68

The majority of participants tested negative for HIV. Most of the participants did not have chronic illnesses. They had previously used contraception, mainly the injectable, as presented in Table 2.

**TABLE 2:** Pre-insertion health status of participants.

Assessment elements	Frequency (*n*)	Percentage
**HIV status**		
Positive	9	7.89
Negative	105	92.11
**Do you have a chronic illness?**		
Yes	6	4.84
No	118	94.35
**Use of previous contraception**		
No	6	4.84
Yes	118	94.35
**Type of previous contraception**		
Condom	8	10.67
Injectable	52	69.33
Pill	9	12.00
IUCD (loop)	3	4.00
Combined methods	3	4.00
**Reasons for opting for implant**		
Long-term contraception	72	60.00
Child spacing	21	17.50
Life planning	8	6.67
Dual protection (use of another contraceptive in addition to the male condom)	5	4.17
Minimise clinic visits	5	4.17
Advised by healthcare worker	1	0.83
Advised by partner	1	0.83
Advised by friend or relative	7	5.83

HIV, human immunodeficiency virus; IUCD, intrauterine contraceptive device.

In Table 3, although the majority of participants did not attend the 3-month check-up postinsertion of the implant, they were told about the side effects of the implant and did experience side effects. Most of the participants were counselled about three side effects, namely heavy bleeding, weight gain and discomfort, pain or headache.

**TABLE 3:** Assessment of 3-month follow-up post-insertion of the implant.

Attendance of 3-month check-up	Frequency (*n*)	Percentage
Yes	33	26.61
No	77	62.10
Not sure	14	11.29
**Were you told about side effects of the implant before insertion?**		
Yes	93	75.00
No	26	20.97
Not sure	5	4.03
**If yes, which side effect?**		
Heavy bleeding	24	24.49
Discomfort, pain, headache	12	12.24
Weight gain	6	6.12
All of the above	35	35.71
Heavy bleeding and weight gain	9	9.18
Heavy bleeding and discomfort, pain, headache	2	2.04
Weight gain and discomfort, pain, headache	6	6.14
Miscellaneous and others	4	4.08
**Did you experience side effects?**		
Yes	104	83.87
No	18	14.52
Not sure	2	1.61
**How long have you had the implant?**		
Less than 6 months	6	4.88
6–11 months	18	14.63
12–23 months	51	41.46
24–35 months	48	32.02
**Reasons for early removal of the implant**		
Heavy bleeding	33	28.45
Discomfort, pain, headache	32	27.59
Weight gain	13	11.21
Wanting to be pregnant	17	14.66
Pressure from husband	2	1.72
Heard a bad story about the implant from relatives or friends	19	16.38

The majority of participants were in the age group 15–20 years when they first started using the etonogestrel subcutaneous contraceptive implant, and it was not the first time that they were using a contraceptive. Most participants did not have the etonogestrel implant inserted after an abortion or after giving birth (Table 4).

**TABLE 4:** Target groups suitable for implant use during pre-insertion counselling.

Target groups	Frequency (*n*)	Percentage
**Age at first implant insertion.**		
15–20 years	61	49.19
21–30 years	52	41.94
39–40 years	11	8.87
**When the implant was inserted, was it the first time that you had ever used contraceptives?**		
Yes	53	42.26
No	71	57.26
**Did you insert the implant after an abortion?**		
Yes	32	25.81
No	92	74.19
**Did you insert the implant after giving birth?**		
Yes	18	14.52
No	106	85.48

## Discussion

In this study, 81% of the participants were between 18 and 29 years of age. This is consistent with other studies carried out in Ethiopia and South Africa.^11,12,20^ In contrast, a study carried out in the United States of America found the implant discontinuation rate to be greatest amongst adolescents aged 14–19 years and lowest amongst women aged 20–45 years.^3^ This inconsistency in the findings of the two studies is because of the fact that the present study did not include participants below 18 years of age because of ethical considerations. This can be considered a limitation of the study.

Other studies in South Africa had findings similar to those of the current study, with the majority of women who removed the implant having a secondary level of education or less.^11,12^ Other consistencies between the current study and other South African studies are that the majority of participants were single, unemployed and had at least one child.^11,12^ The other studies documented the ethnicity of participants, which was omitted from this study.^11,12^ Instead, the religious affinity of participants was documented, and about 94% of the women identified as Christian. This finding is similar to that of a study conducted in the Eastern Cape, South Africa.^11^

The majority of the participants in this study knew their HIV status and were negative (92%). This is different from findings in other studies, where a significant number of women using the implant in South Africa did not know their HIV status, which raised the need to integrate HIV, AIDS and contraception services in South Africa.^10,14,18^ About 69% of the participants in the study had used injectable contraceptives before using the subcutaneous contraceptive implant, and this is consistent with findings from another study in KZN.^12^ Further studies indicate that there is a high and increasing unmet need for modern contraceptives in sub-Saharan Africa and that short-term methods are the type of contraceptive most commonly used in Africa.^8,12^ The finding in this study that most participants were motivated to choose the subcutaneous contraceptive implant because of their desire to have long-term contraception further supports findings that LARCs are often not chosen because of a lack of accurate knowledge to make this decision.^8,14^

In 2011, recommendations for the optimal use of the implant were published by its manufacturer, which included a 3-month post-insertion check-up.^2^ However, despite these recommendations, many of the participants in the current study did not attend the 3-month check-up post-insertion of the implant. Studies in Ethiopia have indicated decreased implant discontinuation rates with regular follow-up after implant insertion.^7,19,20^ The reason that most participants did not attend this check-up might be because the WHO declared that the 3-month check-up post-insertion of the implant was not mandatory.^21^ Also, it is not policy in South Africa to follow up at three months after insertion of the implant, and the South African National Department of Health states that patients should attend follow-up at their own discretion.^22^

Many of the participants in this study removed the implant after one year of use (51; 41.4%) as presented in Table 3, citing side effects as the main reason. This was consistent with what was found in the Eastern Cape,^12^ and the most common side effects encountered in this study were bleeding abnormalities and hormonal complications, which is consistent with the findings of other studies carried out in South Africa.^11,12^

Consistent with recommendations that the implant should be used as a first-line contraceptive for all women who are first-time contraceptive users, including teenagers and young adult females, women post-abortion and vaginal or caesarean section birth, the majority of participants in this study were aged between 18 and 29 years, and it was the first time that they had used the implant.^6,18^ Studies have found that women with a previous history of abortion are more likely to discontinue the implant earlier than prescribed.^21^ This is consistent with the findings in the current study, where 26% of participants inserted the implant after an abortion. This is also mirrored by a study in the Eastern Cape that found that 22% of women requesting early implant removal had a prior history of abortion.^11^

## Conclusion

Women removing the etonogestrel subcutaneous contraceptive implant early at a Pretoria CHC tend to be young women, unemployed, Christian, with a basic or secondary level of education, who have already given birth to at least one child. All participants attended the etonogestrel subcutaneous contraceptive implant pre-insertion counselling services but not the post-counselling services. Heavy bleeding was the main reason for the early removal of the etonogestrel subcutaneous contraceptive implant at the Pretoria CHC.

### Recommendations

The current study has shown a high rate of early removal of the etonogestrel subcutaneous contraceptive implant at Soshanguve 3 CHC secondary to bleeding abnormalities. The 3-month post-insertion check-up would have addressed the management of side effects, as well as the effectiveness^2,23^ of the implant, and thus possibly could have reduced the rate of early removal. In order to establish the association between the lack of a 3-month postinsertion check-up and early removal of the implant, further studies on the topic are encouraged.

Despite the WHO and the Health Ministry of South Africa’s recommendation not to enforce a 3-month post-insertion CHC visit related to the contraceptive implant, this current study has raised concerns about the need for and importance of this follow-up visit.

### Strengths

This data was obtained directly from participants, and no parents or guardians were involved as they could have influenced the participants’ responses. No other study was previously conducted in this study setting.

### Limitations

This study was conducted in only one CHC at a particular point in time; because of this, the findings cannot be generalised to the entire Soshanguve community.

Participants under 18 years of age were excluded due to consent issues.
